# Effect of the conditional cash transfer program *Oportunidades* on vaccination coverage in older Mexican people

**DOI:** 10.1186/1472-698X-13-30

**Published:** 2013-07-08

**Authors:** Aarón Salinas-Rodríguez, Betty Soledad Manrique-Espinoza

**Affiliations:** 1 Center for Evaluation Research and Surveys, National Institute of Public Health, Cuernavaca, Morelos, Mexico

**Keywords:** Older people, Vaccination coverage, Conditional cash transfer, Propensity score matching

## Abstract

**Background:**

Immunization is one of the most effective ways of preventing illness, disability and death from infectious diseases for older people. However, worldwide immunization rates are still low, particularly for the most vulnerable groups within the elderly population. The objective of this study was to estimate the effect of the *Oportunidades* -an incentive-based poverty alleviation program- on vaccination coverage for poor and rural older people in Mexico.

**Methods:**

Cross-sectional study, based on *2007 Oportunidades Evaluation Survey*, conducted in low-income households from 741 rural communities (localities with <2,500 inhabitants) of 13 Mexican states. Vaccination coverage was defined according to three individual vaccines: tetanus, influenza and pneumococcal, and for complete vaccination schedule. Propensity score matching and linear probability model were used in order to estimate the *Oportunidades* effect.

**Results:**

12,146 older people were interviewed, and 7% presented cognitive impairment. Among remaining, 4,628 were matched. Low coverage rates were observed for the vaccines analyzed. For *Oportunidades* and non-*Oportunidades* populations were 46% and 41% for influenza, 52% and 45% for pneumococcal disease, and 79% and 71% for tetanus, respectively. *Oportunidades* effect was significant in increasing the proportion of older people vaccinated: for complete schedule 5.5% (CI_95%_ 2.8-8.3), for influenza 6.9% (CI_95%_ 3.8-9.6), for pneumococcal 7.2% (CI_95%_ 4.3-10.2), and for tetanus 6.6% (CI_95%_ 4.1-9.2).

**Conclusions:**

The results of this study extend the evidence on the effect that conditional transfer programs exert on health indicators. In particular, *Oportunidades* increased vaccination rates in the population of older people. There is a need to continue raising vaccination rates, however, particularly for the most vulnerable older people.

## Background

The current concept of immunization for older people (OP) can be traced back to at least 40 years, when the first influenza vaccination was recommended [1]. However, a renewed interest in this and other vaccines for the high-risk elderly population group has been observed lately. Recent concern stems from three decisive factors: (1) the increasing number of OP in contemporary societies, (2) the high risk of complications and mortality linked to viral influenza and tetanus infections, and (3) the fact that complications caused by these infections can be successfully reduced through immunization. While the basic OP vaccination schedule is characterized by diversity [1,2], most efforts involve vaccines that counterbalance effectiveness with low risk profiles, and recommendations center mainly on a combination of three: tetanus-diphtheria toxoid, influenza and pneumococcal vaccines.

### Tetanus-diphtheria toxoid

Despite its limited number of cases, tetanus continues to be one of the leading causes of various health problems among OP. In 2008, there were 122 cases reported in Mexico of tetanus-diphtheria. From these, 23% pertained to this group with a case-fatality rate of approximately 51% [3]. Evidence indicates that 60% of the reported cases that year occurred after a wound caused by a fall or injury, events which are recurrent among OP. Additionally, approximately 30% of the cases were associated with chronic wounds or underlying medical conditions, such as skin ulcers, abscesses, or gangrene [3].

### Influenza and pneumococcal disease

Influenza is a major communicable disease among OP, particularly among those with chronic respiratory and cardiac conditions. During 2008, older adults aged 65 years and above accounted for 16% of hospitalizations and 63% of the deaths attributable to influenza- and pneumococcal-associated infections in Mexico, from a total of 129,282 hospitalizations reported that year [3]. In fact, the estimated influenza-associated mortality rates during epidemics are within a range of 15/100,000 among OP without high-risk conditions and 400/100,000 among those with one or more high-risk conditions, such as diabetes and chronic pulmonary disease [3].

Pneumococcal pneumonia among OP is two times more frequent than the overall incidence of pneumococcal pneumonia in Mexico [3]. Additionally, infectious diseases, particularly influenza and pneumonia, are among the leading causes of death among OP both in Mexico and worldwide [2-6]. This problem is particularly relevant in designing interventions targeting OP, as the presence of infectious diseases can precipitate the functional impairment process in OP.

Given that many of these diseases are preventable by effective vaccination, high infectious disease burdens and consequent morbidity and mortality among the OP is unwarranted. In fact, many studies have demonstrated that immunization is one of the most effective ways of preventing illness, disability and death from infectious diseases [7-16]. The World Health Organization estimates that OP vaccination reduces the risk of serious complications or death by 70% to 85% [17]. However, despite strong recommendations for influenza and pneumococcal vaccinations, immunization rates are still low globally. Although coverage is gradually increasing in several countries, it is still suboptimal, especially for pneumococcal vaccinations and among some minority groups [5,6,18,19]. Again, this indicates that insufficient vaccination coverage is common among vulnerable OP populations, particularly among the poor ones.

### Conditional cash transfer program

#### Oportunidades

Conditional cash transfer (CCT) programs are one of the most promising approaches to reducing extreme poverty [20-22]. Originally called *Progresa*, *Oportunidades* began in 1997 as a national CCT program intended to reduce extreme poverty in Mexico, and now is one of the largest CCT programs in the world. The government initially targeted rural areas and then extended the Program to urban areas. Currently, *Oportunidades* has enrolled nearly five million families in all 32 states nationwide.

*Oportunidades* provides cash transfers to poor families contingent on their adherence to activities determined by the program, such as: a) school enrolment of children age 6–16; b) attendance by an adult at a monthly health seminar and, c) compliance by all family members to schedule preventive health check-ups. These preventive medical checkups are expected to promote health and welfare to its beneficiaries. This means that each and every one of the residents of the beneficiary household must comply with doctor visits. Depending on the age of the home dweller, these visits are programmed differently. For older adults, these visits should be carried out once every six months. To ensure the compliance of beneficiary households, medical service providers verify the completion of the required health care visits.

The process by which *Oportunidades* selects households that receive the benefits of the program is described in detail in the literature [23], so we will only mention it here in summary. The program was implemented based on a very detailed targeting process aimed at reaching the poorest population in rural areas and avoiding local political influence in designating program beneficiaries.

Targeting of poor households was implemented centrally at the *Oportunidades* headquarters in Mexico City and entailed three stages. First, all localities in the country were ranked using a “marginality index” constructed from 1990 National Census data; this index was stratified into five categories and localities in the bottom categories (high and very high levels of marginality) are pre-selected to be part of the program. Out of 200,151 localities in Mexico, 76,098 rural localities (14.8 million people) were identified in 1997 as having high or very high marginality levels and thus pre-selected for the program.

In the second stage the program identified poor households within the targeted localities. A community census was administered to all households in the selected localities to retrieve information about household characteristics that determine poverty status, including household income, which is used to identify households below the official poverty line. Predicted poverty status was then computed using the results from a discriminant analysis of the poverty indicator that selects the household characteristics that best discriminate between poor and non-poor households. In general, the best predicting variables were a dependency index (number of children to number of working age adults), an overcrowding index (persons per bedroom), the sex, age and schooling of the household head, the number of children, dwelling characteristics such as dirt floor, bathroom with running water, and access to electricity; and possession of durable goods such as a gas stove, a refrigerator, a washing machine and a vehicle. These characteristics were used to compute the discriminant score that separated eligible and non-eligible households in the selected localities.

In stage three, the list of potential beneficiaries of the program was presented to a community assembly where the composition of the list was reviewed; if the assembly rejected a household in the list or an omitted household was alleged to be poor, an administrative process was implemented and the central office delivered a final decision [23].

Once households have been selected -and with the purpose to promote health and welfare of its beneficiaries- *Oportunidades* provides for free the Basic Package of Health Services (*Paquete Básico de Servicios de Salud*). This package is designed to meet the health needs of each population group (children under five, women of childbearing age, older people, etc.). For older people, the package includes the following services: health promotion, nutrition, prevention and control of diseases, sexual and reproductive health, and vaccination schedule. For the present evaluation, our main reference for proper procedures regarding the provision of health services to OP was derived from the *Oportunidades Program Rules of Operation* published in 2007 [24] which is based in the Official Mexican Rule for vaccination schedules [25]. Both focus their basic OP vaccination schedules on tetanus-diphtheria, influenza and pneumococcal immunization.

Based on information from the OP vaccination schedule provided by the program, the objective of this study was to estimate the effect of *Oportunidades* on the vaccination coverage for poor and rural OP in Mexico. Our central hypothesis was that the effect of *Oportunidades* on EP vaccination coverage could reside in the preventive checkups that the program has made mandatory for program eligibility.

## Methods

### Design and sampling

As part of its monitoring and evaluation efforts, the *Oportunidades* program has been conducting evaluation surveys since it was established in 1997. After ten years of formal evaluation in rural areas of Mexico, the program carried out the survey *Encuesta de Evaluación de los Hogares Rurales 2007* (*ENCEL*-*2007*), including, for the first time, a specific module for collecting data on the sociodemographic characteristics, the health status and the living conditions of OP beneficiaries in Mexico. Within this survey, the term older people referred to individuals aged 65 years and over. Information was also gathered on their household demographic, social and economic characteristics. Both datasets (individual older people and household levels) served to analyze the effect of *Oportunidades* on OP vaccination coverage as described in more detail later in the statistical analysis section.

### Data collection and measures

Data was collected through household interviews conducted by trained personnel from the National Institute of Public Health (*Instituto Nacional de Salud Pública*, INSP) of Mexico using standardized survey materials. Interviewers, who were neither detailed on the study’s objectives or hypotheses nor given any questions on program participation, assumed that the study was aimed at evaluating the overall living conditions of OP. All households in the communities visited were surveyed, and those with at least one resident aged 65 years and above were included in the study. Every OP dwelling in these households were interviewed. We used an adapted version of Folstein’s *Mini*-*Mental State* test [26] to identify those who could not respond because of cognitive impairment.

In the case of cognitive impairment, a proxy, defined as the OP’s caregiver, was asked to respond to the questionnaire for the elderly individual, provided that the caregiver was explicitly recognized by the OP as his/her caregiver. Caregivers were also designated as proxies in the case of visual or hearing impairment or any disability which could have prevented the OP from taking the *Mini*-*Mental State* test.

#### Outcomes

The dependent variable selected for the study was the OP self-reported immunization status regarding tetanus, influenza and pneumococcal vaccines. Based on the Official Mexican Norm for vaccination schedules [25], three dichotomous variables were defined. Regarding tetanus and pneumococcal disease, the variable equaled one when the OP had been vaccinated at least once in the past five years, and zero, otherwise. As for influenza, the variable equaled one when the OP had been vaccinated in the past 12 months. These three dichotomous variables were analyzed separately. Another dichotomous variable was created which equaled one if the OP had received the vaccination complete schedule (three vaccines), and zero, otherwise.

#### Treatment variable

Program records on household transfers were used to construct a dichotomous variable for identifying which OP resided in *Oportunidades* beneficiary households, which equaled one if OP resided in a beneficiary household, and zero, otherwise.

#### Covariates

On reviewing literature [27-32] in search of covariates, the following main determinants were identified for OP vaccination: sex, age, functional dependence, chronic disease, paid employment, marital status, educational level, health insurance, socioeconomic level and access to health services. Relevant data was obtained under *ENCEL*-*2007*, as it included a household-level questionnaire apart from the one applied at the individual level to OP. The survey also included a community-level questionnaire on access to health services that provided the following information: the density of nurses, physicians and health units, and the travel time (hours) to the nearest health unit. Both were incorporated into the statistical models to estimate the effect of *Oportunidades* on OP vaccination coverage. Lastly, socio-economic community development data was obtained from a widely used index in Mexico: the *marginalization index*, which was designed and rendered operational by the National Population Council [33].

### Statistical analysis

#### Propensity score matching

Being the first time that data was gathered specifically on OP within the states constituting the original *Oportunidades* evaluation design, the study relied on cross-sectional information, with consequent limitations in its capacity to assess the program’s causal effects. However, it was possible to estimate the effect of the program on the basis of information from *Oportunidades* beneficiary and non-beneficiary households. With *ENCEL*-*2007* having been implemented throughout the households of the communities visited, it was possible to identify which OP belonged to beneficiary households and which OP did not within the same communities. Once this identification step was completed, the intervention sample was established as the group of OP residing in *Oportunidades* beneficiary households, and the control sample as those residing in non-beneficiary households.

It is important to note that this definition of the control group was necessary because of the original evaluation design of *Oportunidades*. Original design did not include the population of older adults as an interest group in which to evaluate *Oportunidades* effect, so that a module to measure the specific health issues of the older people was not included in any of the previous evaluation surveys, until 2007. In this sense, the strategy used in this study was to identify the households that were in 2007 currently receiving program benefits, and within the same localities identify households at the time that were not beneficiaries of the program. In such a way, that the intervention and control groups were defined according to the status of membership in the program they had during the fieldwork ENCEL 2007.

Given the probable incidence of numerous heterogeneous characteristics among the two groups, particularly in regards to their affiliation to *Oportunidades*, the study applied the *Propensity Score Matching* technique [34] to estimate the impact of the program on OP vaccination coverage. We first constructed a propensity score that estimated the probability of enrollment in the Oportunidades program given a set of predictors, and we then created a control group (not-enrolled) and a treatment group (enrolled) having similar propensity scores. We used a logistic regression model to estimate the conditional probability of *Oportunidades* enrollment given a set of covariates, and the caliper matching algorithm allowed us to match, one-to-one, enrolled and not-enrolled EP with similar propensity scores [35,36].

To ensure comparability, we tested the balancing property on pre-treatment covariates between *Oportunidades* enrollees and people not enrolled in the program [37]. We followed the algorithm suggested by Dehejia and Wahba [38,39] to find the best model specification. The method involved the use of different specifications until we obtained a balanced distribution of the following covariates at the individual, household and community levels: health insurance, possession of five items within the household (TV, refrigerator, gas stove, automobile, stereo, and dirt floor), household with children ≤ 13 years, household crowd index, education level of household head, and community deprivation index. Furthermore, we estimated the percent balance of improvement between treated and control groups, which is defined as 100((|**a**| − |**b**|)/|**a**|), where *a* is the balance before and *b* is the balance after matching. Prior research indicated that these matching procedures lead to results similar to those obtained under the randomized experimental design built into the first stage of the *Oportunidades* program [40].

For each of the three dependent variables selected for the study (tetanus, influenza and pneumococcal vaccination) and for the dichotomous variable that defines the complete schedule vaccination, we used a separate linear probability models [41], which produces results comparable to those obtained under the *logit* or *probit* models, alternate procedures for analyzing dichotomous variables. The linear probability model provides the advantage that its coefficients can be directly interpreted in terms of a change in the probability of the event of interest caused by the exposure variable. As the standard error estimators under the linear probability model are heteroskedastic we used robust standard errors [42]. All analyses were performed under the statistical packages R 2.15 [43] and Stata 12.0 [44].

### Ethical review

The Research and Ethics Committees of Mexico’s National Institute of Public Health (*Instituto Nacional de Salud Pública*) approved the *Oportunidades* evaluation. Participants received a detailed explanation of the procedures and signed an informed consent declaration before data collection occurred.

## Results

**Figure 1:**
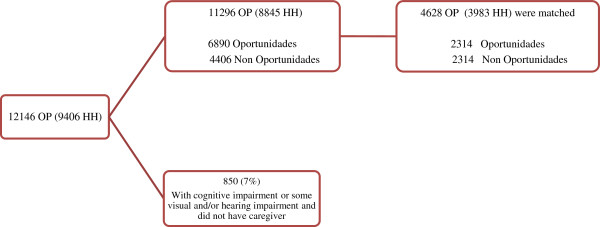
**Population study and analytical sample.** Abbreviations: OP: Older People; HH: Household.

A total of 741 rural communities were visited within 13 states in Mexico. In these communities, was carried out a census of all households, and we identified 42,800 households, for which we obtained a response rate of 91.3%, which means that 44,000 households were surveyed; and of these, 9,406 included at least one OP. In these households each and every one of the OP over 65 years was interviewed. Altogether, 12,146 OP were interviewed, of whom 7% (850 OP) were excluded because they displayed cognitive impairment or other disabilities and did not have caregivers. From the remaining, 4628 were matched (Figure 1).

Table 1 contains OP, household and also some community characteristics. Overall, the data reveals a marginal and vulnerable population with low vaccination coverage rates and a high prevalence of functional dependence, chronic illnesses and illiteracy. It also indicates acute poverty and low medical insurance levels. Table 1 also shows data for full sample and the matched sample.

**Table 1:** Descriptive characteristics for full sample and matched sample

	**Full sample**			**Matched sample**		
	**Non Oportunidades** **n = 4406**	**Oportunidades** **n = 6890**	**p-value**^ⱡ^	**Non Oportunidades** **n = 2314**	**Oportunidades** **n = 2314**	**p-value**^ⱡ^
**Outcomes**						
Complete vaccination schedule	27	32	<0.001	27	34	<0.001
H. Influenzae	41	46	<0.001	40	47	<0.001
Pneumococcal	45	52	<0.001	44	52	<0.001
Tetanus	71	79	<0.001	71	77	<0.001
**Covariates**						
Female	48	48	0.796	48	48	0.906
Age	73.88 (0.11)	73.64 (0.09)	0.097	73.90 (0.15)	73.95 (0.15)	0.828
Indigenous	21	33	0.006	22	22	0.971
Chronic disease (at least one)	50	47	0.007	50	48	0.167
Married or cohabiting	61	63	0.218	60	61	0.489
Paid employment	34	33	0.414	34	33	0.755
literate (*able to read* &*write*)	0.39 (0.01)	0.34 (0.01)	<0.001	37	36	0.246
Have functional dependence	28	29	0.204	28	30	0.144
Six monthly medical check-up	12	29	<0.001	12	29	<0.001
OP with health insurance	36	57	<0.001	43	43	0.970
Time-to-health-unit-service (hrs.)	0.34 (0.01)	0.34 (0.01)	0.802	0.35 (0.01)	0.33 (0.01)	0.191
Locality nurses/doctors density^ⱡⱡ^	1.23 (0.02)	1.25 (0.02)	0.560	1.28 (0.03)	1.19 (0.02)	0.022
Locality doctor offices density^ⱡⱡ^	0.56 (0.01)	0.60 (0.02)	<0.001	0.58 (0.01)	0.58 (0.01)	0.707
Household Asset index	−0.30	−0.40	0.003	−0.50	−0.47	0.630

Cells are percentages or means.

Standard errors in parenthesis.

ⱡⱡ Number per 1000 inhabitants.

ⱡ z-proportion test or mean t-test.

Table 2 shows the results of the logistic regression model to predict affiliation to the program. In general, all variables used were significant (except for OP sex), especially those related to the household assets and demographic structure. Once we estimated the propensity score, we used caliper algorithm (distance = 0.0001) to match one OP residing in an *Oportunidades* household beneficiary with one OP in a non-beneficiary household. The results of the matching process were rigorously evaluated to ensure homogeneity in the observed characteristics, except, of course, regarding program participation. In the matched samples, the differences between both groups were considerably smaller for most of the variables. In fact, there were no significant differences for variables at each of the seven propensity score blocks evaluated. In Additional file 1: Table S1a shows balance before and after matching as well as the percent balance of improvement between treated and control groups, meanwhile Additional file 1: Figure S1a shows the propensity score distribution before and after matching.

**Table 2:** **Logistic regression model to predict affiliation to *Oportunidades *program**

	**Odds ratio**	**Standard error**	**p**-**value**
Sex (female)	1.05	0.06	0.394
Indigenous	1.37	0.08	<0.001
Older people with health insurance	2.27	0.10	<0.001
Household with TV	1.37	0.08	<0.001
Household with refrigerator	0.86	0.05	0.010
Household with gas stove	0.78	0.05	<0.001
Household with auto	0.50	0.04	<0.001
Household with children < = 13	1.61	0.08	<0.001
Household crowd index (2nd quartile)	1.54	0.09	<0.001
Household crowd index (3erd quartile)	1.61	0.10	<0.001
Household head schooling (incomplete primary)	0.85	0.04	<0.001
Household head schooling (complete primary)	1.18	0.11	0.013
Household head schooling (complete secondary)	0.75	0.10	0.093
Household with dirt floor	1.20	0.06	<0.001
Household with stereo	1.30	0.07	<0.001
Locality deprivation index (2nd quartile)	1.24	0.07	<0.001
Locality deprivation index (3erd quartile)	1.19	0.08	<0.001

Vaccination coverage among *Oportunidades* and non-*Oportunidades* populations amounted to 46% and 41% for influenza, 52% and 45% for pneumococcal disease, and 79% and 71% for tetanus, respectively for all samples. Similar numbers were observed for the matching sample (Table 1). After controlling for covariates, results indicate that *Oportunidades* exerts a significant and positive impact on the immunization of OP program beneficiaries. An effect of 0.069 (CI_95%_: 0.038, 0.096; *p* < 0.001) was observed on the influenza vaccine. Under the aforementioned linear probability model, this means that influenza immunization coverage was almost 7% higher among *Oportunidades* OP beneficiaries than among OP non-beneficiaries. Pneumococcal and tetanus vaccine results were analogous with coefficient values of 0.072 (CI_95%_: 0.043, 0.102; *p* < 0.001) and 0.066 (CI_95%_: 0.041, 0.092, *p* < 0.001), respectively. Lastly, the program increased the probability of receiving the complete vaccination schedule (coefficient = 0.055, CI_95%_: 0.028, 0.082; *p* < 0.001) (Table 3).

**Table 3:** **Effect of *Oportunidades *on vaccination coverage in older Mexican people**

	**Coefficient**	**Standard error**	***p***-***value***
Complete vaccination schedule	0.055	0.014	<0.001
H. Influenzae	0.069	0.015	<0.001
Pneumococcal	0.072	0.015	<0.001
Tetanus	0.066	0.013	<0.001

Adjusted for covariates in Table 1.

### Sensitivity analysis

In order to check the robustness of our results, we have carried out a number of alternative analyzes in which we modified the distance within the caliper algorithm. Originally we used a distance = 0.0001, and one control for each treated unit. In subsequent analyzes, we specified three different distances (0.0001, 0.0005, and 0.001) and a different number of treated and control units. These same analyzes were repeated using different ways to sort our observations. First ordering observations according to the unique identifier of household used in the survey, and second according to the household assets index. The results of these analyzes are shown in Table 4. In general, the estimate of the *Oportunidades* effect does not show great variations, and in all cases it is highly significant for complete vaccination schedule and for each of the three individual vaccines.

**Table 4:** Sensitivity analysis

	**Full sample**			**Matched sample**											
	**Ordinary least squares**			**Caliper (one-to-one, distance = 0.0001)ⱡ**			**Caliper (distance = 0.0001)ⱡ**			**Caliper (distance = 0.0005)ⱡ**			**Caliper (distance = 0.001)ⱡ**		
	**Non Oportunidades**	**Oportunidades**		**Non Oportunidades**	**Oportunidades**		**Non Oportunidades**	**Oportunidades**		**Non Oportunidades**	**Oportunidades**		**Non Oportunidades**	**Oportunidade**	
	**n = 4406**	**n = 6890**	**p**	**n = 2314**	**n = 2314**	**p**	**n = 3073**	**n = 4035**	**p**	**n = 3390**	**n = 5463**	**p**	**n = 3473**	**n = 5468**	p
	**β**	**S.E.**		**β**	**S.E.**		**β**	**S.E.**		**β**	**S.E.**		**β**	**S.E.**	
Complete vaccination schedule	0.040	0.001	***	0.055	0.014	***	0.055	0.012	***	0.046	0.012	***	0.045	0.012	***
H. Influenzae	0.046	0.011	***	0.069	0.015	***	0.060	0.012	***	0.053	0.013	***	0.050	0.013	***
Pneumococcal	0.055	0.011	***	0.072	0.015	***	0.068	0.013	***	0.066	0.013	***	0.065	0.013	***
Tetanus	0.068	0.009	***	0.066	0.013	***	0.066	0.011	***	0.053	0.012	***	0.049	0.012	***
							**Caliper (distance = 0.0001)¥**			**Caliper (distance = 0.0005)¥**			**Caliper (distance = 0.001)¥**		
							**Non Oportunidades**	**Oportunidades**		**Non Oportunidades**	**Oportunidades**		**Non Oportunidades**	**Oportunidades**	
							**n = 3073**	**n = 4035**		**n = 3390**	**n = 5463**		**n = 3473**	**n = 5468**	
							**β**	**S.E.**		**β**	**S.E.**		**β**	**S.E.**	
Complete vaccination schedule							0.045	0.014	***	0.046	0.012	***	0.045	0.012	***
H. Influenzae							0.058	0.015	***	0.053	0.013	***	0.050	0.013	***
Pneumococcal							0.065	0.015	***	0.066	0.013	***	0.065	0.013	***
Tetanus							0.068	0.013	***	0.053	0.012	***	0.049	0.012	***

Effect of *Oportunidades* on vaccination coverage in older Mexican people.

Adjusted for covariates in Table 1.

ⱡ Observations sort by unique household/individual identifier.

¥ Observations sort by household assets index.

## Discussion

Since late 1990, CCT programs have been implemented and evaluated in Latin America and the Caribbean. The main feature of such programs is the provision of monetary support and investment in human capital, and in return the beneficiaries must meet certain actions that promote good health and nutrition, and increase their educational levels.

Much of the empirical support on the impact of CCT programs on health and nutrition comes from studies evaluating *Oportunidades*. However, several systematic reviews on the impact of CCT programs, have found an increase in the use of health services for diverse populations (prenatal, children <5 years, children 6–17 years and adults ≥ 18 years), and a decrease on diarrhea in children, and the percentage of mothers who reported illness in children [45,46].

In another vein, the impact of CCT programs on the prevalence of vaccination has been studied primarily in children. For example, the *Familias en Acción* program (Colombia) increased the probability that children have complied with the DPT vaccination schedule [47], while the *Asignación Familiar* program (Honduras) showed an increase in immunization coverage of DPT-Pentavalent in children less than three years [48]. Meanwhile, the *Red de Protección Social* (Nicaragüa) led to large increases in complete schedule vaccination coverage [49]. Finally, the *Oportunidades* program showed a positive effect on the application of BCG in children aged 12–23 months and an increase in vaccination coverage against measles in children 12–23 months [50].

Our study, on the other hand, is part of the global evaluation of the *Oportunidades* program and is based on a large sample of poor and rural elderly persons. To the best of our knowledge, it constitutes the first empirical effort aimed at investigating the association between CCT program participation and vaccination coverage for OP. According to the results, the participation of households with OP enrolled in *Oportunidades*, one of the largest CCT programs in the world, entailed a significant increase in the immunization coverage for the elderly with regard to tetanus, pneumococcal and influenza vaccines, and the complete vaccination schedule. It should be noted, however, that the immunization rates for this population continue to lag, particularly when compared to the standards established under the 2007–2012 Mexican National Health Plan [51], where a goal of at least 85% coverage was set for the basic OP vaccination schedule.

With respect to the hypothesis of our study, we believe that the program's effect is not only due to the mandatory six-monthly check-ups for the OP, but could be related to the fact that beneficiaries of the program have experienced a learning process regarding the use of services health, and the benefits that entails for health [52].

Additionally, the results of this study support the mounting evidence on the short-term benefits of CCT programs for various health indicators in low- and middle-income countries [21,53-55]. However, an important aspect that is omitted from the literature and should be addressed by future research is an analysis investigating the factors mediating the relationship between *Oportunidades* program participation and vaccination coverage specifically and access to health services for the OP population in general. For instance, it is important to determine what roles other government institutions in Mexico (Ministry of Health and the National Institute of Older Adults) play in vaccination coverage. Further, beyond overall program participation, it is important for future research to identify specifically how the obligatory medical checkups required by *Oportunidades* affect vaccination.

Our results suggest that interventions to increase household income could increase vaccination coverage among OP. This could be an argument to promote interventions consisting in conditional cash transfers or non-contributory pension schemes for OP, which is expected to increase older people’s economic and physical autonomy. Autonomy is an essential component of older people’s well-being, to the extent that the World Health Organization in its *Active ageing*[56] policy proposes that it should be considered a key element of programs aimed at this population group.

Several limitations can be noted in our study. First, despite the rigorous matching methods applied to minimize the possibility of an OP selection bias regarding program participation, the causal inferences in the conclusions are not as powerful as they would have been had the study been executed under a purely experimental design. Notwithstanding, it is worth mentioning that another *Oportunidades* study with a methodology comparable to ours confirmed that analogous results are obtained under a quasi-experimental and a wholly experimental approach [40].

Second, the study should be considered as a conservative estimate of program effects. Seeking to minimize selection bias, the original sample size and, consequently, the power of the study were cut down. Nonetheless, the final matched sample size (2314 OP in each group, *Oportunidades* and *Non*-*Oportunidades*) allowed for detecting differences of up to 3.5 percentage points with a power of 90%.

Third, the self-reported vaccination status by the OP or his/her caregiver was estimated as the measure of the outcome variable. Again, while some studies have demonstrated the validity of self-report vaccination among various OP populations [57-59], the question arises as to whether these study populations were rural and extremely poor as well. A study should therefore be conducted to verify if the validity of self-reporting remains applicable in our case.

Fourth, the data associated with the *Oportunidades* evaluation study in rural areas of Mexico was collected in 2007, just two years before the 2009 H1N1 influenza pandemic, which changed the public awareness of vaccination and the health literacy. In fact, for example, Mexico now has the highest rate of vaccination against influenza among OECD countries [60]. The implications of this for our evaluation results are not clear; but the program impact may be even greater since the program's beneficiaries, mostly poor, have been a target population to which efforts have been directed to increase vaccination coverage against influenza.

Lastly, although the safety, effectiveness and cost-effectiveness of OP vaccination schedules have been documented [61], the direct and significant impact of vaccination on specific OP physical health indicators is still pending analysis.

## Conclusions

The program of conditional cash transfer *Oportunidades* has an important effect in increasing vaccination rates in older people residing in rural areas of Mexico.

## Competing interests

The authors declare that they have no competing interests.

## Authors’ contributions

ASR BME had overall responsibility for the project and drafted the manuscript. ASR and BME participated in all phases of the design and implementation and were responsible for the design of survey questionnaires. ASR conducted the data analysis. All authors read and approved the final manuscript.

## Pre-publication history

The pre-publication history for this paper can be accessed here:

http://www.biomedcentral.com/1472-698X/13/30/prepub

## Supplementary Material

Additional file 1 **Estimation and evaluation of the propensity score. Table 1a**. Distribution of variables before and after matching. **Figure 1a**. Propensity score distribution before and after matching. Click here for file
