# Supplementary material for: Effect of the conditional cash transfer program Oportunidades on vaccination coverage in older Mexican people
Source: BMC Int Health Hum Rights. 2013 Jul 8;13:30. doi: 10.1186/1472-698X-13-30 (PMC3711738; doi:10.1186/1472-698X-13-30)
Supplement: Additional file 1 — Estimation and evaluation of the propensity score. Table 1a. Distribution of variables before and after matching. Figure 1a. Propensity score distribution before and after matching. [file 1472-698X-13-30-S1.pptx]

## Slide 1
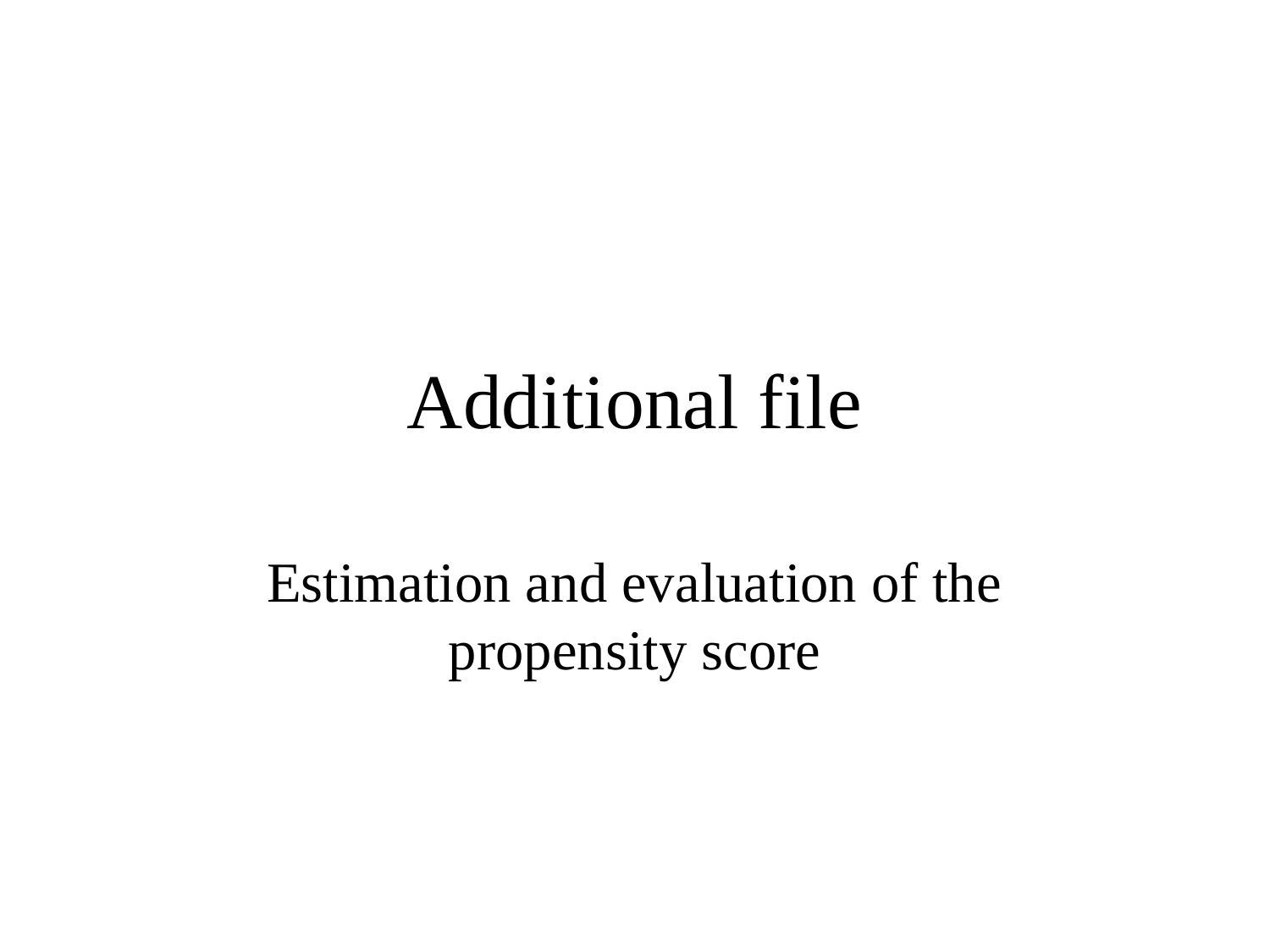

# Additional file
Estimation and evaluation of the propensity score

## Slide 2
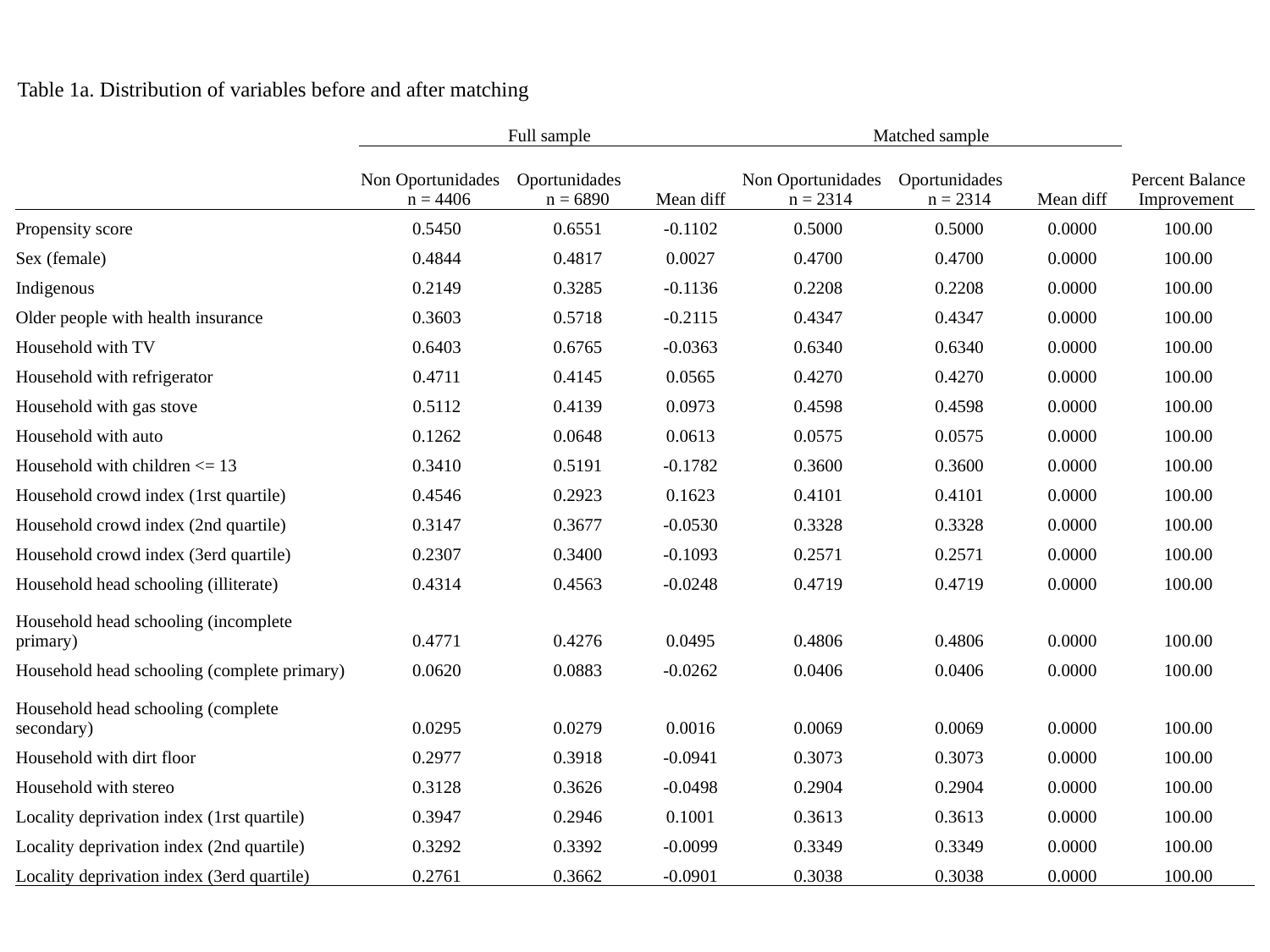

Table 1a. Distribution of variables before and after matching
| | Full sample | | | Matched sample | | | Percent Balance Improvement |
| --- | --- | --- | --- | --- | --- | --- | --- |
| | Non Oportunidades n = 4406 | Oportunidades n = 6890 | Mean diff | Non Oportunidades n = 2314 | Oportunidades n = 2314 | Mean diff | |
| Propensity score | 0.5450 | 0.6551 | -0.1102 | 0.5000 | 0.5000 | 0.0000 | 100.00 |
| Sex (female) | 0.4844 | 0.4817 | 0.0027 | 0.4700 | 0.4700 | 0.0000 | 100.00 |
| Indigenous | 0.2149 | 0.3285 | -0.1136 | 0.2208 | 0.2208 | 0.0000 | 100.00 |
| Older people with health insurance | 0.3603 | 0.5718 | -0.2115 | 0.4347 | 0.4347 | 0.0000 | 100.00 |
| Household with TV | 0.6403 | 0.6765 | -0.0363 | 0.6340 | 0.6340 | 0.0000 | 100.00 |
| Household with refrigerator | 0.4711 | 0.4145 | 0.0565 | 0.4270 | 0.4270 | 0.0000 | 100.00 |
| Household with gas stove | 0.5112 | 0.4139 | 0.0973 | 0.4598 | 0.4598 | 0.0000 | 100.00 |
| Household with auto | 0.1262 | 0.0648 | 0.0613 | 0.0575 | 0.0575 | 0.0000 | 100.00 |
| Household with children <= 13 | 0.3410 | 0.5191 | -0.1782 | 0.3600 | 0.3600 | 0.0000 | 100.00 |
| Household crowd index (1rst quartile) | 0.4546 | 0.2923 | 0.1623 | 0.4101 | 0.4101 | 0.0000 | 100.00 |
| Household crowd index (2nd quartile) | 0.3147 | 0.3677 | -0.0530 | 0.3328 | 0.3328 | 0.0000 | 100.00 |
| Household crowd index (3erd quartile) | 0.2307 | 0.3400 | -0.1093 | 0.2571 | 0.2571 | 0.0000 | 100.00 |
| Household head schooling (illiterate) | 0.4314 | 0.4563 | -0.0248 | 0.4719 | 0.4719 | 0.0000 | 100.00 |
| Household head schooling (incomplete primary) | 0.4771 | 0.4276 | 0.0495 | 0.4806 | 0.4806 | 0.0000 | 100.00 |
| Household head schooling (complete primary) | 0.0620 | 0.0883 | -0.0262 | 0.0406 | 0.0406 | 0.0000 | 100.00 |
| Household head schooling (complete secondary) | 0.0295 | 0.0279 | 0.0016 | 0.0069 | 0.0069 | 0.0000 | 100.00 |
| Household with dirt floor | 0.2977 | 0.3918 | -0.0941 | 0.3073 | 0.3073 | 0.0000 | 100.00 |
| Household with stereo | 0.3128 | 0.3626 | -0.0498 | 0.2904 | 0.2904 | 0.0000 | 100.00 |
| Locality deprivation index (1rst quartile) | 0.3947 | 0.2946 | 0.1001 | 0.3613 | 0.3613 | 0.0000 | 100.00 |
| Locality deprivation index (2nd quartile) | 0.3292 | 0.3392 | -0.0099 | 0.3349 | 0.3349 | 0.0000 | 100.00 |
| Locality deprivation index (3erd quartile) | 0.2761 | 0.3662 | -0.0901 | 0.3038 | 0.3038 | 0.0000 | 100.00 |

## Slide 3
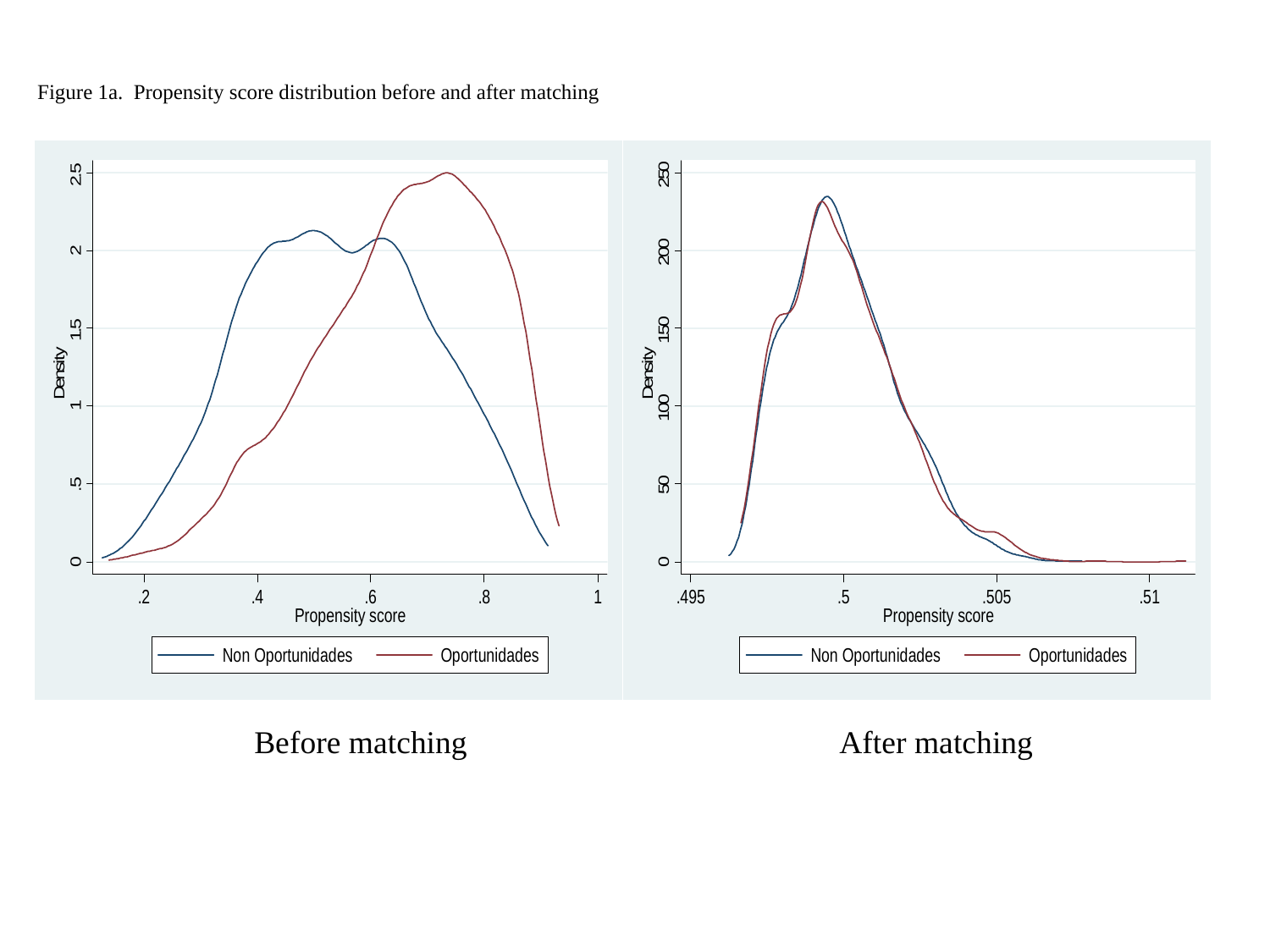

Figure 1a. Propensity score distribution before and after matching
 Before matching			After matching
